# Volatile organic compound analysis, a new tool in the quest for preterm birth prediction—an observational cohort study

**DOI:** 10.1038/s41598-020-69142-4

**Published:** 2020-07-22

**Authors:** Lauren Lacey, Emma Daulton, Alfian Wicaksono, James A. Covington, Siobhan Quenby

**Affiliations:** 1 Warwick Medical School, University of Warwick, Coventry, CV4 7AL UK; 2 Department of Obstetrics and Gynaecology, University Hospitals Coventry and Warwickshire, Coventry, CV2 2DX UK; 3 School of Engineering, University of Warwick, Coventry, CV4 7AL UK

**Keywords:** Translational research, Diagnostic markers

## Abstract

Preterm birth is the leading cause of death worldwide in children under five years. Due to its complex multifactorial nature, prediction is a challenge. Current research is aiming to develop accurate predictive models using patient history, ultrasound and biochemical markers. Volatile organic compound (VOC) analysis is an approach, which has good diagnostic potential to predict many disease states. Analysis of VOCs can reflect both the microbiome and host response to a condition. We aimed to ascertain if VOC analysis of vaginal swabs, taken throughout pregnancy, could predict which women go on to deliver preterm. Our prospective observational cohort study demonstrates that VOC analysis of vaginal swabs, taken in the midtrimester, is a fair test (AUC 0.79) for preterm prediction, with a sensitivity of 0.66 (95%CI 0.56–0.75) and specificity 0.89 (95%CI 0.82–0.94). Using vaginal swabs taken closest to delivery, VOC analysis is a good test (AUC 0.84) for the prediction of preterm birth with a sensitivity of 0.73 (95%CI 0.64–0.81) and specificity of 0.90 (95%CI 0.82–0.95). Consequently, VOC analysis of vaginal swabs has potential to be used as a predictive tool. With further work it could be considered as an additional component in models for predicting preterm birth.

## Introduction

Globally, preterm birth is the leading cause of death in children under 5 years with 15 million babies being born before 37 weeks’ gestation each year^1^. Spontaneous preterm birth is a syndrome, which can be precipitated by a variety of factors (or combination of these) including; infection, inflammation, vascular disease and uterine overdistention^2^. The multifactorial complex nature of spontaneous preterm birth makes its prediction a challenge. A number of factors are needed to activate one or more of the common pathways required for myometrial contractions leading to the premature onset of labour, these include a maternal susceptibility, and an infective, inflammatory, ischaemic or mechanical (over distention) insult^3^. Increasingly research is being carried out to try to improve the predictive value of various tests for preterm birth, including the development of prognostic models that account for both the maternal susceptibility and the insult, using patient history, ultrasound findings and biomarkers^4–8^. First, this aims to enable earlier interventions to facilitate targeted prevention strategies. Secondly to permit better prediction of those patients whom will deliver preterm, to assist in timely administration of treatments to optimise neonatal outcomes, whilst reducing their unnecessary administration as they are not without ramifications^9,10^.

The association between bacterial vaginosis (BV) detected using laboratory techniques and preterm labour has been recognised for many years^11^. Its presence, detected in early pregnancy is associated with an increased risk of preterm labour^11^. Despite this, treatment of BV with antibiotics has not been demonstrated to prevent preterm birth as demonstrated by a recent randomised controlled trial (RCT) and a preceding systematic review and meta-analysis^12,13^. However, this trial focused on the low risk population for spontaneous preterm birth,^12^ and the meta-analysis had substantial heterogeneity (I^2^ = 48%)^13^, therefore treatment of the high risk population for BV with antibiotics to reduce the risk of preterm birth could warrant further investigation due to numerous factors contributing to the onset of preterm labour in a susceptible individual.

A characteristic of bacterial vaginosis is a lack of lactobacilli and an increase in other organisms^11^. More recently molecular-based technologies have allowed for the description of the bacterial component of the vaginal microbiome. These molecular-based techniques have found that a vaginal microbiome with reduced *Lactobacillus* spp. abundance and increased bacterial diversity is associated with preterm delivery^14–18^ and that this can be detected prior to the onset of symptoms^14^.

The mutualistic association between the vagina and the microbiota is a fine balance under the influence of many factors, including sex steroid hormones^19–23^, sexual activity^24,25^ and hygiene practices^25,26^.

Volatile organic compound (VOC) analysis is a technique to monitor microbiota and its metabolic activity, providing insight into the interactions between microbiota and their host. It provides a non-invasive and potentially rapid means to monitor the microbiota and host response. In recent years, VOCs have been demonstrated as a non-invasive diagnostic biomarkers for several diseases including inflammatory bowel disease^27–29^, cancers^30,31^, sepsis^32^ and diabetes^33^. With recent developments in VOC detection and analysis technology it now has the potential to be realistically translated into clinical practice.

In some cases preterm labour is thought to be the result of both vaginal flora and host response to this flora^34^. Thus, for the prediction of preterm labour, a test that both detects differences in the vaginal flora and the host epithelial response to these differences could be clinically useful.

Previous studies have demonstrated promise for the diagnosis of BV using VOC technology^35,36^. However, much of the previous VOC detection and analysis work deployed instruments which had several limitations for the clinical setting including the need for excessive training, substantial costs, significant data analysis, and concerns with reproducibility of results, consequently these instruments were not amenable to become a rapid point of care test at that time^37^. In our study we aimed to build on this work and assess whether novel VOC technology could detect BV and thus reflect vaginal flora. We aimed to identify if specific patterns of VOCs from vaginal swabs taken in pregnancy, were associated with preterm delivery in a population of pregnant women attending a preterm prevention clinic due to pre-existing risk factors for preterm birth using VOC detection and analysis technology involving machine learning, which with development has the potential to be translated into clinical practice and overcome the previously discussed concerns.

### Approvals

The study was approved by the NHS Research Ethics Committee West Midlands Birmingham South on 14^th^ January 2014 and sponsored by the University of Warwick. Approval from University Hospitals Coventry & Warwickshire Research & Development was acquired on 17^th^ January 2014. All participants gave written informed consent. The study was funded by the Biomedical Research Unit in Reproductive Health, University of Warwick. Research was carried out according to The Code of Ethics of the World Medical Association (Declaration of Helsinki).

### Participants

The population was a prospective observational cohort of repeated sampling of 216 patients between weeks 10–29 of pregnancy (total 493 sets of swabs were taken, with at least 2 swabs per patient), from women attending the high-risk preterm prevention clinic at a tertiary level teaching hospital from January 2017-August 2018. Women were excluded from analysis relating to preterm delivery if they required iatrogenic preterm delivery (n = 11) or delivery data were not available (n = 9). This left a remaining cohort of 196 women with spontaneous onset of labour at a known gestation. Demographic data including age at booking pregnancy, BMI, ethnicity, smoking status and indication for attendance to the preterm prevention clinic were collected about each woman (Table 1). An individualised management plan was made for each patient at their first visit, this was subject to modification dependent upon transvaginal ultrasound cervical length measurements. Some patients required cervical cerclage (history or ultrasound indicated) and other patients used vaginal progesterone pessaries. Pregnancy outcome was obtained from clinical notes (Table 1). If patients were detected to have bacterial vaginosis using standard care, this was treated with a five day course of 2% clindamycin cream.

**Table 1:** Baseline characteristics.

Characteristic	BV positive (n = 26)	BV negative (n = 190)	P value	Term (≥ 37 + 0 weeks) (n = 157)	Preterm (≤ 36 + 6 weeks) (n = 39)	P value
**Age**						
Mean (SD)- yrs	31.0 + /− 4.8	31.2 + /− 4.9	0.8666	31.6 ± 5.0	29.8 ± 4.4	0.0267*
Distribution-no. (%)						
< 35 yrs	17 (65.4)	142 (74.7)		111 (70.7)	34 (87.2)	
≥ 35 yrs	9 (34.6)	48 (25.3)		46 (29.3)	5 (12.8)	
**BMI at booking**^a^						
Median (IQR)-kg/m^2^	25.0 (22.5–33.0)	25.3 (22.2–29.4)	0.5074	25.3 (22.4–29.3)	25.3 (21.8–31.2)	0.8101
Distribution-no. (%)						
< 30 kg/m^2^	18 (69.2)	142 (77.6)		119 (75.8)	29 (74.4)	
≥ 30 kg/m^2^	8 (30.7)	41 (22.4)		34 (21.7)	10 (25.6)	
**Ethnicity**^b^–**no. (%)**						
White	21 (80.8)	145 (76.3)	0.4597	124 (79.0)	33 (84.6)	0.0608
Black	4 (15.4)	20 (10.5)		19 (12.1)	0 (0)	
Asian	1 (3.8)	15 (7.9)		12 (7.6)	4 (10.3)	
Other	0	10 (5.3)		2 (1.3)	2 (5.1)	
**Smoking in pregnancy-no. (%)**						
Yes	4 (15.4)	21 (11.1)	0.8029	18 (11.5)	8 (20.5)	0.2015
No	20 (76.9)	152 (80.0)		127 (80.9)	30 (76.9)	
Not known	2 (7.7)	17 (8.9)		12 (7.6)	1 (2.6)	
**Indication for attending clinic-no. (%)**						
Previous preterm ≥ 24 wk to < 34 wk				50 (31.9)	30 (76.9)	< 0.0001*
Previous midtrimester loss < 24 wk				38 (24.2)	4 (10.3)	
History of cervical surgery				69 (43.9)	5 (12.8)	
**Gestation at delivery**						
Midtrimester miscarriage	1	1		–	2	
Extreme preterm (< 28 weeks)	2	4		–	6	
Very preterm (28–31 + 6 weeks)	3	8		–	10	
Late preterm (32–36 + 6 weeks)	7	12		–	21	
Term (≥ 37 weeks)	9	149		157	–	
Not known	3	6		–	–	
Iatrogenic preterm delivery	1	10		–	–	

This includes 216 patients attending the preterm prevention clinic who had bacterial vaginosis diagnosed in pregnancy versus those who did not, and the 196 patients including who delivered preterm versus term (excluding those who had iatrogenic preterm delivery or if delivery information was not available). Pregnancy outcomes of patients recruited to the study from the preterm prevention clinic are illustrated.

^a^BMI of 7 women in BV negative group not known and BMI of 4 women in term group not known.

^b^Patient reported.

## Results

### Chemical analyser

**Figure 1:**
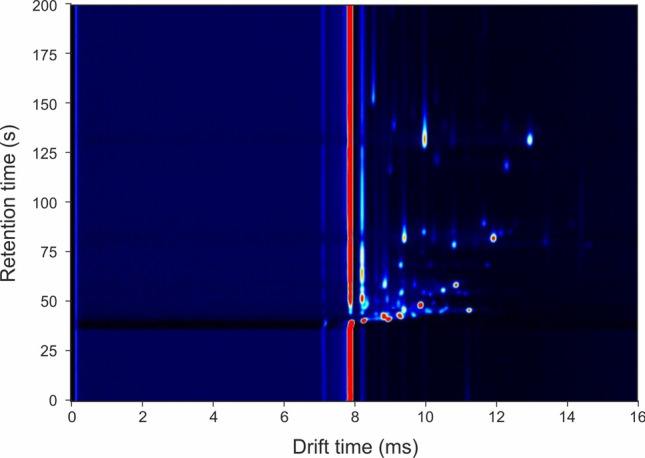
Typical GC-IMS output to a vaginal swab. The x-axis refers to IMS drift time and the y-axis the retention time of chemicals eluding from the GC column.

Figure 1 shows a typical output from the GC-IMS instrument for a preterm labour swab. The x-axis shows the drift time within the IMS and the y-axis the retention time of the GC (the time taken for the molecule to elude from the column). In Fig. 1, the background is dark blue, with the remaining colours being chemicals detected by the IMS. It also shows intensity with the red being the most abundant ions. The majority of the chemicals are seen as ‘circles’ on the output. The long red line in the y-axes is the systems response when there are no chemicals present. As can be seen, there is significant chemical information within the sample.

### Sample group

Between January 2017 and August 2018, 493 vaginal swabs were taken from 216 women in pregnancy during their attendance to a preterm prevention clinic and birth outcome data collected. All women were asymptomatic for preterm birth. Their demographics are illustrated in Table 1.

Tests of normality were performed on the data, if data was identified to be parametric, students t t-tests was used and mean reported, if data was non-parametric Mann–Whitney U test were used for statistical analysis and median reported. For categorical variables chi square test was used.

As can be seen from Table 1 the baseline demographics were comparable between the two groups (with BV positive/BV negative or delivered term/delivered preterm) with the exception of maternal age in those women who delivered term compared to preterm. Those who delivered preterm were statistically significantly younger than those women who delivered at term. The indications for attendance to the clinic differed between the two groups. The number of women with a BV positive vaginal swab during pregnancy in our cohort was 26 (12.0%). The number of women who delivered preterm in our cohort was 39, corresponding to 19.9% of this high-risk population. 9.2% of deliveries were before 32 weeks gestation. Of the 23 women who had a positive test for BV in pregnancy (and delivery data known), 13 went into spontaneous preterm labour (one was delivered preterm for iatrogenic reasons), corresponding to 59.1% (13/22). Of the women who had negative test for BV in pregnancy 14.4% (25/174) delivered preterm.

### Statistical results

**Figure 2:**
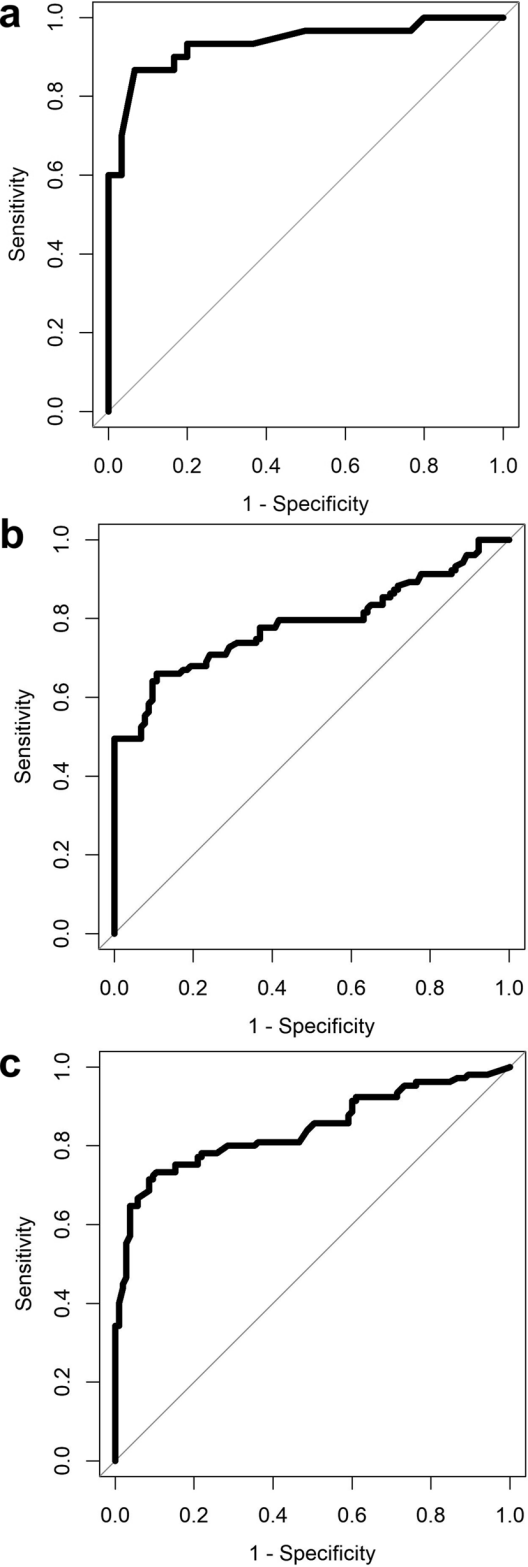
Receiver operator characteristic curves for VOC analysis of vaginal swabs for prediction of BV (**a**) and prediction of preterm labour (**b**, **c**) before 37 + 0 gestation from asymptomatic patients at an increased risk of spontaneous preterm birth for preterm delivery. (**b**) first swab taken in pregnancy (**c**) swab taken closest to delivery. (**a**) illustrates that VOC analysis is an excellent test for the diagnosis of BV with an AUC of 0.94. (**b**) demonstrates that taking a swab in the midtrimester and analysis of VOCs is a test which is fair for the subsequent prediction of preterm labour with an AUC of 0.79. (**c**) shows that VOC analysis of a swab taken later in pregnancy (late second or early third trimester) is a good test for the prediction of preterm birth with an AUC of 0.84.

The data from the VOC detection instruments was analysed as described in the methods section and the statistical output is shown in Table 2. This table also illustrates the gestation the swabs were taken in pregnancy and the number of days between when the swabs were taken and delivery. The ROC curves are illustrated in Fig. 2.

**Table 2:** Statistical outputs for VOC analysis for the prediction of BV and preterm delivery from vaginal swabs taken in pregnancy.

Classifier	Random forest	Gaussian process	
**Statistical output (95%CI) for prediction of bacterial vaginosis**			
AUC	0.92 (0.84–1)	0.94 (0.87–1)	
Sensitivity	0.83 (0.65–0.94)	0.87 (0.69–0.96)	
Specificity	0.97 (0.83–1)	0.93 (0.78–0.99)	
Positive predictive value	0.96	0.93	
Negative predictive value	0.85	0.88	
*p* value (between groups)	< 0.001	< 0.001	

	Delivered preterm	Delivered term	*p* value
**First swab taken in pregnancy**			
Gestation when swab taken (weeks), Median (IQR)	17 + 4 (15 + 5 – 21 + 3)	16 + 6 (15 + 5 – 20 + 1)	0.3849
Number of weeks between swab taken and delivery, Median (IQR)	14 + 4 (10 + 1 – 17 + 1)	22 + 2 (19 + 1 – 24 + 0)	< 0.0001

Classifier	Random forest	Gaussian process	
**Statistical output (95%CI) for prediction of preterm delivery before 37 weeks**			
AUC	0.79 (0.72–0.85)	0.73 (0.66–0.80)	
Sensitivity	0.66 (0.56–0.75)	0.67 (0.57–0.76)	
Specificity	0.89 (0.82–0.95)	0.76 (0.66–0.84)	
Positive predictive value	0.86	0.73	
Negative predictive value	0.72	0.70	
*p* value (between groups)	< 0.001	< 0.001	

	Delivered preterm	Delivered term	*p* value
**Swab taken closest to delivery**			
Gestation when swab taken (weeks), Median (IQR)	25 + 2 (22 + 6 – 27 + 1)	22 + 3 (18 + 5 – 25 + 5)	0.0019
Number of weeks between swab taken and delivery, Median (IQR)	7 + 2 (3 + 5 – 11 + 1)	17 + 1 (13 + 0 – 21 + 0)	< 0.0001

Classifier	Random forest	Gaussian process	
**Statistical output (95%CI) for prediction of preterm delivery before 37 weeks**			
AUC	0.84 (0.79–0.90)	0.77 (0.70–0.83)	
Sensitivity	0.73 (0.64–0.81)	0.68 (0.58–0.76)	
Specificity	0.9 (0.82–0.95)	0.76 (0.67–0.84)	
Positive predictive value	0.88	0.74	
Negative predictive value	0.77	0.70	
*p* value (between groups)	< 0.001	< 0.001	

This table illustrates the gestation when the first swab was taken per patient in pregnancy and when the swab was taken closest to delivery per patient.

From Table 2, the mean gestation the first swab was taken in pregnancy did not differ between those who delivered at term and those who delivered preterm. The swab taken closest to delivery was taken nearer to delivery in the patients who delivered preterm. As expected, there was a shorter number of weeks from when the first swabs and swabs taken closest to delivery were taken in those who delivered preterm to those who delivered term. Table 2 also summarises the predictive statistics calculated using the methods described.

## Discussion

This is the first study examining volatile organic compounds from vaginal swabs for the prediction of BV and preterm birth. As expected, VOC analysis did detect BV as this condition is known to be associated with specific odour. This finding could have some clinical utility in a rapid bedside diagnostic test for BV and this concept is currently being developed by others^38^.

Our results showing that VOCs on vaginal swabs, taken in asymptomatic women attending a preterm prevention clinic, could provide some useful information in predicting preterm labour. As preterm labour is a multifactorial condition the VOC test will only ever be able to predict some preterm births, and this is reflected in our specificity reported (0.65). Others, for example those caused by poor placental function or uterine anomalies are unlikely to be predicted using this test. The AUCs are good for the clinical context in prediction of spontaneous preterm birth in high risk asymptomatic women with the first swabs taken in the second trimester (AUC of 0.79), which improved in the last swabs taken closest to delivery (AUC 0.84). Of importance is that VOCs had a positive predictive value of up to 86% even when sampled 10–24 weeks before the event. This raises the possibility of using VOCs to develop a personalised approach to preterm labour prevention as there is plenty of time between the test and delivery to introduce preventative measures in susceptible individuals. Again, as expected, the nearer to delivery that the swab was taken the better the predictive performance and indeed the second swab taken 3–21 weeks before delivery had negative predictive value of up to 88% which could be useful clinically for planning location of delivery.

In our population, the indication for attendance to the preterm prevention clinic varied between those who delivered preterm or delivered at term. As expected, there was a higher proportion of those with a high-risk risk factor (previous second trimester loss) who went on to deliver preterm and higher proportion of those with an intermediate-risk risk factor (previous cervical surgery) who went on to deliver at term^39^. Nearly two thirds of women who had a positive swab for BV, despite treatment went on to deliver preterm, supporting the previous studies that BV is associated with an increased risk of preterm birth but treatment for BV in pregnancy does not reduce the risk of preterm delivery^12,13^. Future risk stratification using VOCs could include these variables as has been done successfully by others^40^.

More recently cervical length measurement, quantitative fetal fibronectin and clinical factors have been combined and developed into a predictive model for preterm birth in asymptomatic women. This work has demonstrated that spontaneous preterm both can be accurately predicted with AUC values ranging from 0.75 to 0.90^40^. VOCs have potential benefits, in addition to the existing published model for several reasons. Firstly because of the physiological difference between the tests. Quantitative fetal fibronectin is thought to detect inflammation of fetal membranes and together with the cervical length and clinical risk factors in the QUiPP App perhaps suggest cerclage or pessary to preterm prevention^40^. In contrast the VOCs test is thought to detect a combination of the vaginal microbiome and the maternal response hence suggesting treatment to restore the vaginal flora^41^ and reduce the maternal response to this flora perhaps with progesterone^42–44^. Secondly, quantitative fetal fibronectin is limited by the gestation at which it can be measured, after 22 weeks, this is not a limitation that applied to VOCs. Thirdly VOC analysis is likely to be a cheaper test requiring the purchase of a machine then using usual hospital swabs in the sensor rather than having to buy cassettes containing chemical assays per swab. We suggest that the addition VOC analysis to existing models could lead to the development of a test that is the basis of a personalised approach to preterm labour prevention. Much more work is needed to determine the use of VOCs in this field. We plan to compare our VOC analysis to the vaginal microbiome and determine if specific VOCs correlate with bacterial communities.

At this point in time, we do not have the specific biomarkers that resulted in these differences. Furthermore, we do not have any data about how individual patients vaginal VOCs change throughout gestation. In future studies we are planning to collect and analyse swabs throughout pregnancy and closer to delivery and undertake a deeper VOC analyses to understand how these specific VOCs change. Recent work on the vaginal microbiome after 20 weeks gestation has demonstrated the microbiome diversity in both women who deliver preterm and term converges and remains stable for the remaining weeks of pregnancy^45^, supporting the chances that the VOCs detected on our swabs could be similar from the midtrimester onwards. However, this is making the presumption that the host isn’t changing and influencing the VOCs produced. Steroid hormones are known to change throughout pregnancy, they could influence VOCs, which reflect interactions between the microbiome and the host.

In conclusion, this novel work has demonstrated that VOC analysis has the potential to be used as a predictive tool to support the prediction of preterm birth and aid personalised prevention strategies. In the future this could be considered as an additional component to be utilised in models to help solve the multifactorial complex problem of accurate prediction and prevention of preterm delivery.

## Methods

### Test methods

A speculum examination was performed and a vaginal swab taken for microbiology culture and sensitivity testing and for examination under the microscope using the Hay/Ison criteria. This was placed into a nonnutritive transport medium as per routine care. Concurrently, two cotton swabs were used to obtain index test vaginal samples for VOC analysis. The index test swabs were then placed in a universal containers and snap frozen in liquid nitrogen and stored at − 80 °C. Specimens were obtained by gently rotating the swabs across the mucosa of the vagina. Samples were taken in a consecutive series from all women who consented in the clinic, some women consented to samples being taken during every attendance to the clinic.

Swabs were analysed to first to assess whether VOCs could detect BV. Subsequently, the first swab, taken at the earliest gestation per patient was analysed using VOC detection technology to identify if specific VOC patterns could be detected in those who went on to deliver preterm (before 37 + 0 weeks gestation). Then the swab taken closest to delivery per patient was analysed using VOC detection technology to identify if specific VOC patterns predicted preterm birth.

### Chemical analyzer

For chemical vapour analysis, index swabs were shipped to the BioMedical Sensors Laboratory, School of Engineering, University of Warwick. The odours/VOCs emanating from the samples were analysised using G.A.S. GC-IMS instrument (Dortmund, Germany), which is based on Gas Chromatograph – Ion Mobility Spectrometery principles (GC-IMS). This is a highly sensitive odour detection technology that has been previously used by our group in clinical studies analysing a range of biological samples^46,47^. In brief, the GC front end is used to pre-separate molecules based on their interaction with a stationary phase coating on a long column, eluding from the column at different times. These molecules are then ionized (in our case with a tritium sounce) and enter a drift tube IMS. Here, the ions are pushed through a tube by applying an electric field and detected as they exit the tube. Against the flow of sample ions, a buffer gas is pushed through the tube in the opposite direction to the ions. In general, large sample molecules were struck many times and slowed down, whilst smaller sample molecules were struck less and kept their momentum. Therefore, drift time is a function of the interaction between the electric field the buffer gas and the ions. This instrument was chosen over more traditional GCMS, as the basic sensitivity of the instrument is much higher, it can use nitrogen as the carrier gas (so no expensive gas such as helium), has a lower unit cost than GCMS and has a smaller form factor, thus has the potential to be used on a ward setting. However, a limitation of this setup was that we were unable to identify specific VOC biomarkers. Thus, the output of the instrument was analysed using a pattern recognition technique.

### Chemical testing and analysis

Samples were shipped from University Hospitals Coventry and Warwickshire to Warwick University on dry-ice and briefly stored at − 20 °C before testing. Sample preparation comprised of thawing the swab at 4 °C and transferring it to a 20 ml gas vial, which is subsequently sealed with a crimp top lid fitted with a PTFE septum. Prior to measurement samples were heated to 40 °C for 10 min. The sample line for the GC-IMS was inserted into the septa of the vial using a needle and 2 ml of sample was pulled into the analytical platform. The machine settings were as follows: E1: 150 ml/min (for the drift tube IMS), E2: 20 ml/min (for the GC column) and the pump at 25% total power. The total run time was 10 min. The temperatures were set to: T1: 45 °C, T2: 80 °C, and T3: 70 °C.

### Statistical analysis

For data analysis, the data was first extracted from the native file format to a text file using the L.A.V. Software (G.A.S. Dortmund, Germany). Then dimentional reduction steps were used to decrease the number of datapoints and remove the backgound. This is undertaken as the data has high dimensionality (typically 11 million data points per sample), but low information content, with the majority of the data located in the centre of the output (shown in Fig. 1). To reduce the dimensionality of the data, a crop is first applied that reduces the output to the data to this central section. The values of this crop are selected manually by inspection of a large number of samples to ensure that all the chemical information is contained within it. Once selected, the same crop values are applied to all the samples automatically. Then a threshold is used to remove the background, which contains no useful information. Again, this process is undertaken by manual inspection and then the same value applied to all of the samples automatically. These processes reduce the number of non-zero data points by a factor of × 100, which in turn reduces the computation overhead of analyzing the data. Once completed, the data was analysed using a tenfold cross validation. Within each fold, the data was divided into a 90% training set and a 10% test set. Features with discriminary power were identified from the training set using a rank-sum test and 100 features with the lowest *p* value were taken forward for classification. Then, two different classifiers, specifically Random Forest and Gaussian process, were applied. These have previously been found to be good classifiers for GC-IMS data^46,47^. Once the training models have been created, they were applied to the same features in the test set. This process is repeated ten times until all the data has a test result. Utilising this method, we needed to have the same number of index test positive and negative samples, negative samples were chosen at random. This process provided test probabilities for each sample and from this, statistical values, including sensitivity and specificity were calculated.

### Ethics approval

The study protocol was approved by the NHS Research Ethics Committee West Midlands Birmingham South on 14th January 2014 (13/WM/0486).

## Supplementary information

Supplementary Information 1.

Supplementary Information 2.

## Data Availability

The raw demographic data is available in the supplementary material section. Raw data are available to readers but due to the large file sizes these are held on Zenodo via the following link https://zenodo.org/record/3902115#.XvCJ5C2ZNbV

## References

[1] 1World Health Organisation. *Preterm Birth Fact sheet *(2017).

[2] Goldenberg RL, Culhane JF, Iams JD, Romero R (2008). Epidemiology and causes of preterm birth. Lancet.

[3] Romero R, Dey SK, Fisher SJ (2014). Preterm labor: one syndrome, many causes. Science.

[4] Stock SJ (2018). Quantitative fibronectin to help decision-making in women with symptoms of preterm labour (QUIDS) part 1: individual participant data meta-analysis and health economic analysis. BMJ Open.

[5] Son M, Miller ES (2017). Predicting preterm birth: cervical length and fetal fibronectin. Semin. Perinatol..

[6] Esplin MS (2017). Predictive accuracy of serial transvaginal cervical lengths and quantitative vaginal fetal fibronectin levels for spontaneous preterm birth among nulliparous women. JAMA.

[7] Vandermolen BI (2016). Quantitative fetal fibronectin and cervical length to predict preterm birth in asymptomatic women with previous cervical surgery. Am. J. Obstet. Gynecol..

[8] Jwala S (2016). Evaluation of additive effect of quantitative fetal fibronectin to cervical length for prediction of spontaneous preterm birth among asymptomatic low-risk women. Acta Obstet. Gynecol. Scand..

[9] Rodriguez A (2019). Antenatal corticosteroid therapy (ACT) and size at birth: a population-based analysis using the Finnish Medical Birth Register. PLoS Med..

[10] Räikkönen K, Gissler M, Kajantie E (2020). Associations between maternal antenatal corticosteroid treatment and mental and behavioral disorders in children. JAMA.

[11] Lamont RF (2015). Advances in the prevention of infection-related preterm birth. Front. Immunol..

[12] Subtil D (2018). Early clindamycin for bacterial vaginosis in pregnancy (PREMEVA): a multicentre, double-blind, randomised controlled trial. Lancet.

[13] Brocklehurst P, Gordon A, Heatley E, Milan SJ (2013). Antibiotics for treating bacterial vaginosis in pregnancy. Cochrane Database Syst. Rev..

[14] Brown RG (2018). Vaginal dysbiosis increases risk of preterm fetal membrane rupture, neonatal sepsis and is exacerbated by erythromycin. BMC Med..

[15] Brown RG (2019). Establishment of vaginal microbiota composition in early pregnancy and its association with subsequent preterm prelabor rupture of the fetal membranes. Transl. Res. J. Lab. Clin. Med..

[16] Paramel Jayaprakash T (2016). High diversity and variability in the vaginal microbiome in women following preterm premature rupture of membranes (PPROM): a prospective cohort study. PLoS One.

[17] DiGiulio DB (2015). Temporal and spatial variation of the human microbiota during pregnancy. Proc. Natl. Acad. Sci. USA.

[18] Kindinger LM (2016). Relationship between vaginal microbial dysbiosis, inflammation, and pregnancy outcomes in cervical cerclage. Sci. Transl. Med..

[19] Hickey RJ, Zhou X, Pierson JD, Ravel J, Forney LJ (2012). Understanding vaginal microbiome complexity from an ecological perspective. Transl. Res. J. Lab. Clin. Med..

[20] Eschenbach DA (2000). Influence of the normal menstrual cycle on vaginal tissue, discharge, and microflora. Clin. Infect. Dis..

[21] Brotman RM, Ravel J, Bavoil PM, Gravitt PE, Ghanem KG (2014). Microbiome, sex hormones, and immune responses in the reproductive tract: challenges for vaccine development against sexually transmitted infections. Vaccine.

[22] Hyman RW (2012). The dynamics of the vaginal microbiome during infertility therapy with in vitro fertilization-embryo transfer. J. Assist. Reprod. Genet..

[23] Kazi YF, Saleem S, Kazi N (2012). Investigation of vaginal microbiota in sexually active women using hormonal contraceptives in Pakistan. BMC Urol..

[24] Gajer P (2012). Temporal dynamics of the human vaginal microbiota. Sci. Transl. Med..

[25] Schwebke JR, Richey CM, Weiss HL (1999). Correlation of behaviors with microbiological changes in vaginal flora. J. Infect. Dis..

[26] Brotman RM, Ravel J, Cone RA, Zenilman JM (2010). Rapid fluctuation of the vaginal microbiota measured by Gram stain analysis. Sex. Transm. Infect..

[27] Arasaradnam RP (2013). A novel tool for noninvasive diagnosis and tracking of patients with inflammatory bowel disease. Inflamm. Bowel. Dis..

[28] Arasaradnam RP (2016). Non-invasive exhaled volatile organic biomarker analysis to detect inflammatory bowel disease (IBD). Dig. Liver Dis..

[29] van Gaal N (2017). Faecal volatile organic compounds analysis using field asymmetric ion mobility spectrometry: non-invasive diagnostics in paediatric inflammatory bowel disease. J. Breath Res..

[30] Arasaradnam RP (2014). Detection of colorectal cancer (CRC) by urinary volatile organic compound analysis. PLoS ONE.

[31] Arasaradnam RP (2018). Noninvasive diagnosis of pancreatic cancer through detection of volatile organic compounds in urine. Gastroenterology.

[32] van Keulen BJ (2018). Late-onset sepsis in preterm infants can be detected preclinically by fecal volatile organic compound analysis: a prospective, multicenter cohort study. Clin. Infect. Dis..

[33] Esfahani S, Wicaksono A, Mozdiak E, Arasaradnam PR, Covington AJ (2018). Non-invasive diagnosis of diabetes by volatile organic compounds in urine using FAIMS and Fox4000 electronic nose. Biosensors.

[34] Smith SB, Ravel J (2017). The vaginal microbiota, host defence and reproductive physiology. J. Physiol..

[35] Chandiok S (1997). Screening for bacterial vaginosis: a novel application of artificial nose technology. J. Clin. Pathol..

[36] Hay P, Tummon A, Ogunfile M, Adebiyi A, Adefowora A (2003). Evaluation of a novel diagnostic test for bacterial vaginosis: 'the electronic nose'. Int. J. STD AIDS.

[37] Covington JA (2015). The application of FAIMS gas analysis in medical diagnostics. Analyst.

[38] Blankenstein T (2015). Point-of-care (POC) diagnosis of bacterial vaginosis (BV) using VGTest™ ion mobility spectrometry (IMS) in a routine ambulatory care gynecology clinic. Arch. Gynecol. Obstet..

[39] Story L (2019). Reducing the impact of preterm birth: preterm birth commissioning in the united kingdom. Eur. J. Obstet. Gynecol. Reprod. Biol. X.

[40] Watson HA (2019). Development and validation of the predictive models for the QUiPP App vol 2: a tool for predicting preterm birth in high-risk asymptomatic women. Ultrasou. Obstet. Gynecol..

[41] Witkin SS (2015). The vaginal microbiome, vaginal anti-microbial defence mechanisms and the clinical challenge of reducing infection-related preterm birth. BJOG Int. J. Obstet. Gynaecol..

[42] Matei A, Saccone G, Vogel JP, Armson AB (2019). Primary and secondary prevention of preterm birth: a review of systematic reviews and ongoing randomized controlled trials. Eur. J. Obstet. Gynecol. Reprod. Biol..

[43] Medley N, Vogel JP, Care A, Alfirevic Z (2018). Interventions during pregnancy to prevent preterm birth: an overview of Cochrane systematic reviews. Cochrane Database Syst. Rev..

[44] Alfirevic Z, Stampalija T, Medley N (2017). Cervical stitch (cerclage) for preventing preterm birth in singleton pregnancy. Cochrane Database Syst. Rev..

[45] Haque MM, Merchant M, Kumar PN, Dutta A, Mande SS (2017). First-trimester vaginal microbiome diversity: a potential indicator of preterm delivery risk. Sci. Rep..

[46] Rouvroye DM (2019). Faecal scent as a novel non-invasive biomarker to discriminate between coeliac disease and refractory coeliac disease: a proof of principle study. Biosensors.

[47] Mozdiak E, Wicaksono AN, Covington JA, Arasaradnam RP (2019). Colorectal cancer and adenoma screening using urinary volatile organic compound (VOC) detection: early results from a single-centre bowel screening population (UK BCSP). Tech. Coloproctol..

