# Supplementary material for: Volatile organic compound analysis, a new tool in the quest for preterm birth prediction—an observational cohort study
Source: Sci Rep. 2020 Jul 22;10:12153. doi: 10.1038/s41598-020-69142-4 (PMC7376243; doi:10.1038/s41598-020-69142-4)
Supplement: Supplementary file 1 — Supplementary Information 1. [file 41598_2020_69142_MOESM1_ESM.docx]

**Volatile organic compound analysis, a new tool in the quest for preterm birth prediction – an observational cohort study**

**Authors**

Lauren Lacey^1,2*^

Emma Daulton^3^

Alfian Wicaksono^3^

James A. Covington^3^

Siobhan Quenby^1,2^

Supplementary material: Demographic data for study
